# LLM4FB: A One-Sided CSI Feedback and Prediction Framework for Lightweight UEs via Large Language Models

**DOI:** 10.3390/s26020691

**Published:** 2026-01-20

**Authors:** Xinxin Xie, Xinyu Ning, Yitong Liu, Hanning Wang, Jing Jin, Hongwen Yang

**Affiliations:** 1 School of Information and Communication Engineering, Beijing University of Posts and Telecommunications, Beijing 100876, China; xiexinxin@bupt.edu.cn (X.X.); nxybupt@bupt.edu.cn (X.N.); yanghong@bupt.edu.cn (H.Y.); 2 Future Research Lab, China Mobile Research Institute, Beijing 100053, China; wanghanning@chinamobile.com (H.W.); jinjing@chinamobile.com (J.J.)

**Keywords:** massive MIMO, CSI feedback, channel prediction, large language model, industrial IoT

## Abstract

Massive MIMO systems can substantially enhance spectral efficiency, but such gains rely on the availability of accurate channel state information (CSI). However, the increase in the number of antennas leads to a significant growth in feedback overhead, while conventional deep-learning-based CSI feedback methods also impose a substantial computational burden on the user equipment (UE). To address these challenges, this paper proposes LLM4FB, a one-sided CSI feedback framework that leverages a pre-trained large language model (LLM). In this framework, the UE performs only low-complexity linear projections to compress CSI. In contrast, the BS leverages a pre-trained LLM to accurately reconstruct and predict CSI. By utilizing the powerful modeling capabilities of the pre-trained LLM, only a small portion of the parameters needs to be fine-tuned to improve CSI recovery accuracy with low training cost. Furthermore, a multiobjective loss function is designed to simultaneously optimize normalized mean square error (NMSE) and spectral efficiency (SE). Simulation results show that LLM4FB outperforms existing methods across various compression ratios and mobility levels, achieving high-precision CSI feedback with minimal computational capability from terminal devices. Therefore, LLM4FB presents a highly promising solution for next-generation wireless sensor networks and industrial IoT applications, where terminal devices are often strictly constrained by energy and hardware resources.

## 1. Introduction

Multiple-input multiple-output (MIMO) technology is a key enabler for enhancing the spectral efficiency and reliability of wireless communication systems. In frequency-division duplex (FDD) systems, accurate CSI at the base station is essential for effective downlink pre-coding. However, as the number of antennas increases dramatically, traditional codebook-based CSI quantization schemes suffer from excessive feedback overhead and limited quantization accuracy. To overcome these limitations, DL-based CSI feedback methods have been proposed and have demonstrated strong potential [1]. Moreover, deep-learning-based CSI feedback architectures have been proposed, including Transformer backbone networks [2], domain knowledge-guided meta-learning approaches [3], and feature vector designs tailored for pre-coding structures [4]. In order to improve the generalization ability of CSI feedback models under distribution drift, recent studies have also explored the lightweight adaptive framework [5] and unsupervised learning pathway [6]. Most existing methods adopt a two-sided architecture, where a UE-side encoder neural network compresses the CSI, and a corresponding BS-side decoder performs CSI reconstruction. Although the aforementioned methods have yielded notable performance improvements, their encoders impose substantial computational and memory burdens on resource-constrained UEs. This limitation is especially acute in emerging applications like wearable electronics and industrial wireless sensor networks, as low-power sensor nodes cannot sustain the energy cost of complex deep learning inference. Furthermore, the UE-side encoder must be frequently updated to cope with time-varying channel conditions, resulting in poor generalization and additional training overhead.

To address these challenges, a one-sided feedback scheme has been proposed [7]. In this architecture, computationally intensive deep learning models are fully deployed at the BS side, while UE only performs lightweight compression operations (such as linear projection), thereby significantly reducing its computational burden. Beyond explicit compression, an ultra-low-rate implicit CSI feedback scheme which leverages the reciprocity of the bidirectional channel has been developed to further reduce uplink overhead [8]. Hao Luo further employed Type I/II codebooks at the UE side for CSI compression and investigated codebook-independent enhancement methods for deep-learning-based CSI feedback [9]. However, the performance of one-sided frameworks critically depends on the reconstruction capability of the BS-side decoder. Under highly compressed CSI or dynamically varying channel conditions, existing decoders often struggle to recover the CSI with sufficient accuracy.

In recent years, large language models (LLMs) have gained widespread attention for their remarkable capabilities [10]. Their potential in shaping future 6G systems has also been highlighted in relevant surveys [11]. The application of LLMs has long transcended the boundaries of natural language processing and has extended into the field of wireless communications—where several foundational models have already emerged to address various wireless tasks. For example, WirelessGPT introduced a multitask pre-training framework with about 80 million parameters [12], while LLM4WM explored the adaptation of LLMs for wireless multitasking [13]. In channel modeling, ChannelGPT employs a GPT-2 based architecture to tackle long-distance channel prediction and multimodal perception tasks [14]. Motivated by these advances, this work incorporates LLMs into the one-sided feedback framework, leveraging their strong modeling strength to enhance the accuracy of CSI reconstruction and prediction.

Inspired by Liu et al. [15], this paper proposes LLM4FB, a novel framework that integrates a pre-trained large language model (LLM) into the base station (BS) decoder. The core philosophy is to leverage the extensive semantic representations encapsulated within the pre-trained LLM to enhance channel state information (CSI) estimation. Specifically, we employ a parameter-efficient fine-tuning (PEFT) strategy, where the majority of the LLM parameters remain frozen while only specific modules, such as normalization layers, are updated. To further optimize system-level performance, we introduce a composite loss function that jointly minimizes the normalized mean square error (NMSE) and maximizes spectral efficiency (SE).

The proposed LLM4FB framework demonstrates superiority over existing CSI feedback paradigms in three critical aspects: reconstruction fidelity, computational efficiency, and cross-domain generalization.

In terms of reconstruction accuracy, the framework exhibits exceptional robustness, particularly in challenging compression regimes. By treating the coarse pseudoinverse reconstruction as a corrupted sequence and leveraging the LLM’s denoising capabilities, LLM4FB effectively recovers channel semantics. At a non-compressed state (γ=0), the method achieves an NMSE of 0.044, representing a 31% improvement over Transformer-based baselines (0.064) and significantly outperforming CNN (0.051) and LSTM (0.077) architectures. More notably, under extreme compression scenarios (γ=64), where conventional methods experience severe performance collapse—with CNN and Transformer degrading to NMSEs of 0.53 and 0.494, respectively—LLM4FB maintains a resilient NMSE of 0.464.

Regarding parameter efficiency, the proposed fine-tuning strategy drastically mitigates the training overhead typically associated with large-scale models. Unlike conventional approaches necessitating full-parameter updates, LLM4FB restricts optimization to the layer normalization layers, resulting in only 0.97 M trainable parameters out of a total 85.23 M (approximately 2% of model capacity). This reduction translates to a 50-fold decrease in computational cost and memory usage compared to full fine-tuning. Despite this sparse update mechanism, the framework yields a spectral efficiency of 8.494 bps/Hz, approaching the theoretical upper bound of 8.510 bps/Hz achieved by full-parameter training. This finding suggests that the pre-trained weights possess sufficient general feature extraction capabilities, requiring only minimal distribution alignment to adapt to wireless channel characteristics.

Furthermore, the framework offers superior adaptability across diverse propagation environments. While traditional deep learning models often require hundreds of epochs to converge when facing shifts in antenna configurations or channel scenarios (e.g., UMa to UMi), LLM4FB exploits the inherent alignment between the next-token prediction task in NLP and temporal sequence prediction in CSI feedback. Empirical results indicate that cross-scenario fine-tuning converges within 10–50 epochs, achieving an adaptation speed 2–10 times faster than training from scratch. This rapid deployment capability is particularly advantageous for the dynamic environmental requirements of 6G systems.

The main contributions of this paper are as follows:

- A novel one-sided CSI feedback and prediction framework LLM4FB is proposed, which uses a pre-trained LLM to enhance the BS-side decoder capability and achieves high-accuracy CSI prediction for lightweight UEs.
- An efficient parameter fine-tuning strategy is designed, and a multiobjective loss function is proposed that jointly optimizes NMSE and SE, enabling further improvement of system performance.
- Extensive simulations verify the effectiveness of LLM4FB under various compression ratios and moving speeds, and its performance surpasses multiple existing baseline methods.

## 2. System Model

A single-cell multiple-input single-output (MISO) orthogonal frequency-division multiplexing (OFDM) system is considered. The BS is equipped with a dual-polarized uniform planar array (UPA) comprising $N_{t}$ antennas, where $N_{h}$ and $N_{v}$ denote the number of antennas along the horizontal and vertical dimensions, respectively ($N_{t}=2N_{h}N_{v}$). The UE is equipped with a single omnidirectional antenna.

### 2.1. Channel Model

A classical cluster-based multipath channel model is employed. The channel consists of $N_{c}$ scattering clusters, each containing $N_{path}$ propagation paths. The channel vector $h_{k,t}\in C^{N_{t}\times 1}$ at subcarrier *k* and time *t* is given by

(1) $$h_{k,t}=\sum_{c=1}^{N_{c}}\sum_{l=1}^{N_{path}}\alpha_{c,l}e^{j2\pi f_{D,c,l}t}e^{-j2\pi \tau_{c,l}f_{k}}a\left(\phi_{c,l},\theta_{c,l}\right),$$

where $\alpha_{c,l}$, $\tau_{c,l}$, $\phi_{c,l}$, and $\theta_{c,l}$ denote the complex gain, delay, azimuth angle, and elevation angle of the *l*-th path in cluster *c*, respectively. a(ϕ,θ) represents the array steering vector at the BS. The Doppler shift of the *l*-th path in cluster can be calculated by

(2) $$f_{D,c,l}=\frac{vf_{c}}{c}\cos\left(\vartheta_{c,l}\right),$$

with *v* being the UE speed, $\vartheta_{c,l}$ the angle between the UE motion direction and the angle of arrival, *c* the speed of light, and $f_{c}$ the carrier frequency. These expressions reveal that high user mobility introduces significant Doppler spread and temporal variations, posing challenges for accurate CSI prediction.

### 2.2. Signal Transmission Model

During downlink transmission, the BS performs pre-coding based on the available CSI. Let $N_{\mathrm{sc}}$ denote the number of active subcarriers. The received signal at the UE on subcarrier *k* is

(3) $$y_{k,t}=h_{k,t}^{H}w_{k,t}s_{k,t}+n_{k,t},$$

where $s_{k,t}$ is the transmitted symbol, $w_{k,t}\in C^{N_{t}\times 1}$ is the pre-coding vector, and $n_{k,t}∼\mathrm{CN}\left(0,\sigma_{n}^{2}\right)$ denotes additive white Gaussian noise (AWGN). To maximize the downlink rate, matched-filter (MF) pre-coding is employed:

(4) $$w_{k,t}=\frac{{\hat{h}}_{k,t}}{∥{\hat{h}}_{k,t}{∥}_{2}},$$

where ${\hat{h}}_{k,t}$ denotes the BS-side estimate of the true channel $h_{k,t}$. The average spectral efficiency can be represented as

(5) $$R=\frac{1}{N_{\mathrm{sc}}}\sum_{k=1}^{N_{\mathrm{sc}}}E\left[\log_{2}\left(1+\frac{|h_{k,t}^{H}w_{k,t}{|}^{2}}{\sigma_{n}^{2}}\right)\right].$$

From (4) and (5), the quality of ${\hat{h}}_{k,t}$ directly determines the pre-coding performance and, thus, the attainable SE. This underscores the critical importance of accurate CSI feedback and prediction.

### 2.3. CSI Feedback and Prediction Problem Formulation

In FDD systems, CSI feedback is essential for maintaining reliable downlink performance. The overall procedure consists of three stages: CSI compression, feedback, and reconstruction and prediction at the BS. Assume that the UE acquires perfect CSI over $L_{h}$ consecutive time slots, denoted by $H_{\mathrm{past}}=\left[H_{t-L_{h}+1},\ldots ,H_{t}\right]\in C^{N_{\mathrm{sc}}\times L_{h}\times N_{t}}$. The objective is to exploit this historical information to predict the CSI for the subsequent $L_{f}$ time slots at the BS, i.e., $H_{\mathrm{future}}=\left[H_{t+1},\ldots ,H_{t+L_{f}}\right]$.

**(1)**  **Compression at UE:** The UE applies an encoding function $f_{\mathrm{enc}}$ to compress the historical CSI $H_{\mathrm{past}}$ into a low-dimensional representation $y\in C^{M}$, where $M\ll N_{\mathrm{sc}}\times L_{h}\times N_{t}$.
  (6) $$y=f_{\mathrm{enc}}\left(H_{\mathrm{past}}\right).$$
**(2)**  **Feedback:** The compressed vector y is transmitted to the BS over the uplink channel. For analytical clarity, an error-free feedback link is assumed.
**(3)**  **Reconstruction and Prediction at BS:** Upon receiving y, the BS employs a decoding function $g_{\mathrm{dec}}$ to reconstruct the historical CSI and generate predictions for future CSI.
  (7) $${\hat{H}}_{\mathrm{future}}=g_{\mathrm{dec}}\left(y\right).$$
  The system aims to design the encoder $f_{\mathrm{enc}}$ and decoder $g_{\mathrm{dec}}$ such that the discrepancy between the true future CSI $H_{\mathrm{future}}$ and its estimate ${\hat{H}}_{\mathrm{future}}$ is minimized, thereby improving the spectral efficiency.

## 3. LLM-Based One-Sided CSI Feedback Framework

**Figure 1:**
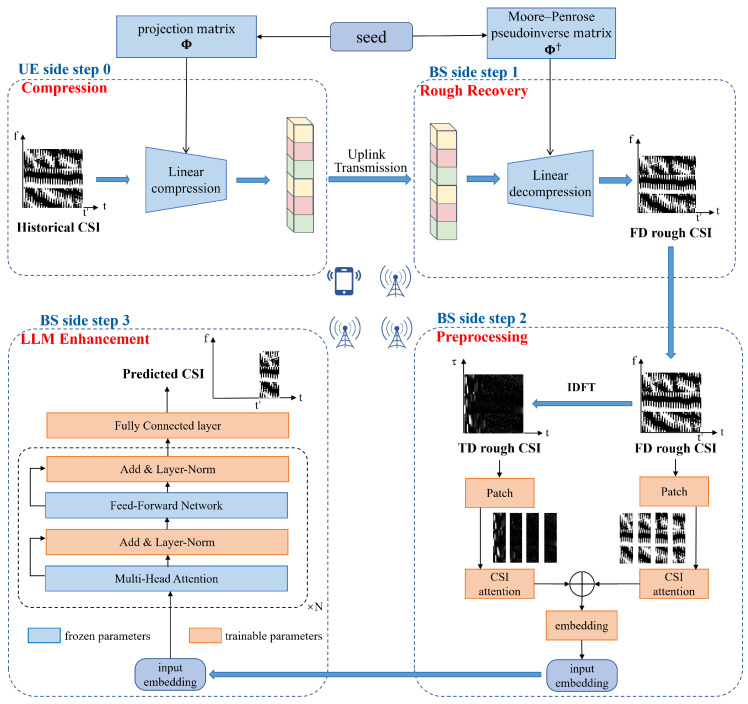
Illustration Overview of the LLM4FB framework: the UE carries out a simple linear compression step, and the BS then performs an initial reconstruction using the pseudoinverse before applying an LLM-based module to refine the CSI and make predictions.

To address the high computational burden and limited generalization capability of conventional two-sided CSI feedback architectures, the LLM4FB framework is developed—a one-sided feedback framework that shifts the major inference workload to the BS. The overall pipeline is depicted in Figure 1.

### 3.1. UE-Side Low-Complexity Compression

Within the LLM4FB framework, the UE performs only a random linear projection to compress CSI, corresponding to a minimalist encoder $f_{\mathrm{enc}}$. For the CSI tensor $H_{\mathrm{past}}\in C^{N_{\mathrm{sc}}\times L_{h}\times N_{t}}$ spanning $N_{\mathrm{sc}}$ subcarriers and $L_{h}$ consecutive time slots, processing is simplified by compressing the per-antenna CSI matrix $H_{i}\in C^{N_{\mathrm{sc}}\times L_{h}}$ independently for each antenna $i=1,\ldots ,N_{t}$. Specifically, the UE vectorizes the real and imaginary parts of $H_{i}$ separately and concatenates them into a real-valued vector of dimension $2N_{\mathrm{sc}}L_{h}$, which is then projected by a random matrix Φ:

(8) $$y_{i}=\Phi \cdot \mathrm{vec}\left(ℜ\left(H_{i}\right),ℑ\left(H_{i}\right)\right),$$

where $\Phi \in R^{M\times (2N_{\mathrm{sc}}L_{h})}$ is the random projection matrix and $y_{i}\in R^{M\times 1}$ denotes the compressed representation to be fed back. The compression ratio is defined as $\mathrm{CR}=(2N_{\mathrm{sc}}L_{h})/M$. In this architecture, the UE avoids storing a large-scale neural network—only retaining the seed used to generate the deterministic realization of Φ, thereby substantially reducing storage requirements.

To ensure reproducibility and theoretical guarantees, the random projection matrix Φ is constructed following specific design principles. Each element of $\Phi \in R^{M\times (2N_{\mathrm{sc}}L_{h})}$ is independently drawn from a standard Gaussian distribution N(0,1):

(9) $$\Phi_{i,j}∼N\left(0,1\right),\;\forall i\in \left\{1,\ldots ,M\right\},j\in \left\{1,\ldots ,2N_{\mathrm{sc}}L_{h}\right\}.$$

This Gaussian ensemble is chosen because it provably satisfies the restricted isometry property (RIP) with high probability. Specifically, for an *S*-sparse channel vector, the matrix preserves Euclidean distances when $M\geq C\cdot S\log(N_{\mathrm{sc}}L_{h}/S)$ for a universal constant *C*, which provides theoretical justification for accurate recovery even under extreme compression.

The pseudoinverse recovery in (10) implicitly normalizes the measurement energy. Although no explicit $1/\sqrt{M}$ scaling is applied during compression, the Moore–Penrose pseudoinverse $\Phi^{†}=\Phi^{T}{\left(\Phi \Phi^{T}\right)}^{-1}$ acts as a matched filter that optimally reconstructs the signal in the least-squares sense, automatically accounting for the energy distribution of the projection.

Critically, the same projection matrix Φ is applied to all $N_{t}$ antennas. This design choice offers two advantages: (1) Preserving spatial coherence: Applying identical linear transformations across antennas maintains the relative phase and amplitude relationships in the compressed domain, enabling the LLM to exploit spatial correlations during reconstruction. (2) Implementation efficiency: Sharing Φ reduces storage overhead from $O(M\cdot N_{\mathrm{sc}}L_{h}\cdot N_{t})$ to $O(M\cdot N_{\mathrm{sc}}L_{h})$, which is crucial for massive MIMO systems with hundreds of antennas. To ensure consistency across experiments, the random seed for Φ generation is fixed, guaranteeing that the BS and UE operate on identical projection matrices.

### 3.2. BS-Side CSI Recovery and LLM Enhancement

The BS-side decoder $g_{\mathrm{dec}}$ is responsible for recovering and predicting CSI from the received compressed vectors $y_{i}$. The recovery process consists of two stages: an initial linear reconstruction step, followed by LLM-based enhancement step.

Upon reception of $y_{i}$, the BS first obtains an initial estimate ${\hat{H}}_{\mathrm{init},i}$ by applying the (Moore–Penrose) pseudoinverse of the projection matrix:

(10) $$\mathrm{vec}\left({\hat{H}}_{\mathrm{init},i}\right)=\Phi^{†}y_{i},$$

where $\Phi^{†}$ denotes the Moore–Penrose pseudoinverse. (When $\Phi \Phi^{T}$ is non-singular, one valid form is $\Phi^{†}=\Phi^{T}{\left(\Phi \Phi^{T}\right)}^{-1}$). As the compression is highly underdetermined ($M\ll 2N_{\mathrm{sc}}L_{h}$), the initial estimate ${\hat{H}}_{\mathrm{init},i}$ inevitably contains substantial reconstruction error and information loss.

To recover high-fidelity CSI from this noisy initial estimate, an LLM-based enhancement module is introduced. By leveraging the expressive prior knowledge embedded in a pre-trained LLM, the module models complex joint time-frequency correlations in the CSI and performs refined reconstruction and prediction. The enhancement module comprises four components—preprocessing, embedding, the LLM backbone, and an output head—which collectively convert the initial real-valued reconstructions into LLM-compatible inputs and generate the final CSI estimates. Detailed architecture and processing steps are described in the following subsections.

#### 3.2.1. Preprocessing and Tokenization Strategy

The initial reconstruction yields the tensor ${\hat{H}}_{\mathrm{init}}$, which comprises complex numbers stored in floating point format. However, LLMs typically operate on discrete tokens, necessitating a mapping from continuous-valued CSI signals to the discrete input space.

In order for large models to better extract time-domain and frequency-domain information, An inverse discrete Fourier transform (IDFT) is performed on the roughly recovered CSI to map the frequency-domain CSI to the delay domain. In the delay domain, the energy of the signal is concentrated in a small number of taps, allowing the model to better learn features and converge quickly.

Inspired by the patch strategy of Vision Transformers [16]: instead of directly feeding all the information into the model at once as typical models do, the delay-time CSI matrix is divided into several non-overlapping patches of size *P*. Each patch captures the local time–frequency characteristics of specific channel regions; after flattening, it is mapped to a latent vector space through a learnable linear projection. This process is similar to tokenization in natural language processing, with each patch acting as a high-dimensional feature describing a segment of the channel state, compressing the length while extracting local features effectively.

Mathematically, given the delay-time CSI matrix ${\hat{H}}_{\mathrm{init},i}^{\mathrm{delay}}\in R^{N_{\mathrm{delay}}\times L_{h}}$ for antenna *i*, we partition it into $S=N_{\mathrm{delay}}/P$ patches $\left\{P_{1},P_{2},\ldots ,P_{S}\right\}$, where each patch $P_{j}\in R^{P\times P}$. Each patch is then flattened and projected into a $d_{\mathrm{model}}$-dimensional embedding space:

(11) $$E_{j}=W_{e}\cdot \mathrm{vec}\left(P_{j}\right)+b_{e},$$

where $W_{e}\in R^{d_{\mathrm{model}}\times P^{2}}$ and $b_{e}\in R^{d_{\mathrm{model}}}$ are learnable parameters. The resulting sequence of patch embeddings $\left\{E_{1},E_{2},\ldots ,E_{S}\right\}$ serves as the input to the LLM backbone.

To preserve the sequential order essential for prediction, we add learnable positional embeddings to the patch embeddings. As standard self-attention is permutation-invariant; it cannot capture the order of the CSI patches on its own. The sinusoidal positional encoding strategy is employed. For a patch at position pos in the sequence and a dimension index 2i or 2i+1 in the embedding space, the positional encoding PE is defined as

(12) $$\begin{array}{cc} PE_{\left(pos,2i\right)} & =\sin\left(\frac{pos}{10,000^{2i/d_{\mathrm{model}}}}\right),\\ PE_{\left(pos,2i+1\right)} & =\cos\left(\frac{pos}{10,000^{2i/d_{\mathrm{model}}}}\right), \end{array}$$

where $d_{\mathrm{model}}$ is the dimension of the embedding vectors. These positional encodings are added element-wise to the patch embeddings before being fed into the LLM backbone.

In our implementation, we set the patch size to P=4 to balance local feature extraction and computational efficiency. Given the historical CSI sequence length $L_{h}=16$ time slots and delay-domain dimension $N_{\mathrm{delay}}=16$, the input delay-time matrix is 16×16. After patch partitioning with stride P=4, we obtain S=16 patches in total (4 patches along the delay axis × 4 patches along the time axis). Each patch $P_{j}\in R^{4\times 4}$ is flattened into a 16-dimensional vector and projected to $d_{\mathrm{model}}=768$ dimensions. After processing through the LLM backbone, these 16 patch embeddings are unpacked and reshaped back to the original 16×16 delay-time structure before final CSI reconstruction. This design preserves spatial locality in both delay and time domains while maintaining a manageable sequence length for the attention mechanism.

#### 3.2.2. Model Architecture Selection and Pre-Training Task Alignment

We adopt the GPT-2 Small architecture [10] as the backbone for LLM4FB, which consists of 12 Transformer decoder blocks with 12 attention heads per layer and a hidden dimension of $d_{\mathrm{model}}=768$. The total parameter count is approximately 117 million. To balance inference efficiency and model capacity for CSI feedback tasks, we truncate the architecture to 6 layers, reducing the total parameters to 85.23 million while maintaining sufficient representational power.

The selection of GPT-2 is motivated by the inherent alignment between its pre-training task and CSI prediction. GPT-2 is pre-trained on a next-token prediction objective, where the model learns to predict the probability distribution $P(x_{t}|x_{<t})$ of the next token $x_{t}$ given the preceding tokens $x_{<t}$. Mathematically, this corresponds to modeling autoregressive conditional dependencies in sequential data. This task is fundamentally congruent with channel prediction, where the objective is to estimate future CSI $H_{t+1:t+L_{f}}$ conditioned on historical observations $H_{t-L_{h}+1:t}$. Both tasks require capturing long-range temporal dependencies and extrapolating patterns beyond observed sequences.

Through the patch embedding strategy described earlier, we convert the continuous-valued CSI signal into a sequence of discrete feature vectors, enabling direct utilization of GPT-2’s learned capabilities in modeling long-range causal dependencies. During pre-training on massive text corpora, GPT-2 develops internal representations that encode sequential patterns, temporal correlations, and contextual reasoning—skills that transfer effectively to wireless channel modeling despite the domain shift from language to radio signals. This cross-domain transferability has been empirically validated in recent works applying LLMs to time-series forecasting [15] and multimodal sensing tasks.

#### 3.2.3. LLM Backbone and Self-Attention Mechanism

The core of our framework is the pre-trained LLM backbone (specifically, a GPT-2 variant), which consists of stacked Transformer decoder blocks. The fundamental operation within these blocks is the Masked Multi-Head Self-Attention (MSA). Mathematically, for an input sequence of CSI embeddings X, the attention mechanism computes three matrices: queries (Q), keys (K), and values (V). The attention score is calculated as

(13) $$\mathrm{Attention}\left(Q,K,V\right)=\mathrm{softmax}\left(\frac{QK^{T}}{\sqrt{d_{k}}}\right)V,$$

where $d_{k}$ is the dimension of the key vectors. In the CSI prediction problem, the attention mechanism allows the model to concentrate on the time points that actually contribute to the prediction. For instance, when the channel exhibits a periodic fading pattern due to a certain scatterer, the model can identify and emphasize earlier samples that share the same trend, which helps it anticipate the upcoming state. This behavior contrasts with RNNs and LSTMs, which process sequences in order and often have difficulty preserving information over long spans. The Transformer overcomes this limitation by attending to the entire sequence context simultaneously. This ability is particularly useful when the channel response is formed by multiple paths with different Doppler shifts.

To reduce training overhead while retaining the LLM’s pre-trained knowledge, a parameter-efficient fine-tuning (PEFT) strategy is adopted, updating only the layer normalization and positional embedding parameters. This allows LLM4FB to exploit a large model capacity while training only a small fraction of the parameters compared to conventional deep learning approaches. The output of the LLM, denoted as $Z_{\mathrm{LLM}}$, represents the enhanced CSI feature embeddings.

### 3.3. Computational Complexity Analysis

A key motivation for the proposed one-sided framework is to offload the computational burden from the UE to the BS. Here, the computational complexity of both ends is analyzed in terms of floating-point operations (FLOPs).

At the UE side, the compression process involves a linear projection of the vectorized channel matrix. Given the input dimension $D_{\mathrm{in}}=2N_{\mathrm{sc}}L_{h}$ and the compressed dimension *M*, the complexity is dominated by the matrix-vector multiplication, which is $O(M\cdot D_{\mathrm{in}})$. As $M\ll D_{\mathrm{in}}$ due to the high compression ratio, this operation is extremely lightweight and can be efficiently implemented on low-power mobile chipsets.

At the BS side, the complexity is primarily determined by the LLM inference. For a Transformer-based model with $L_{\mathrm{layer}}$ layers, hidden dimension $d_{\mathrm{model}}$, and sequence length *S*, the complexity of the self-attention mechanism is $O(S^{2}\cdot d_{\mathrm{model}})$, and the feed-forward network contributes $O(S\cdot d_{\mathrm{model}}^{2})$. Consequently, the total complexity at the BS is approximately $O(L_{\mathrm{layer}}\cdot \left(S^{2}d_{\mathrm{model}}+Sd_{\mathrm{model}}^{2}\right))$. Although this scales quadratically with the sequence length, the BS is typically equipped with high-performance computing resources (e.g., GPUs), making this computational cost acceptable. This asymmetric complexity distribution aligns perfectly with the resource constraints of practical FDD massive MIMO systems.

### 3.4. Output and Loss Function

The enhanced features $Z_{\mathrm{LLM}}$ are mapped back to the original CSI dimension via a linear projection layer, producing the final predicted CSI ${\hat{H}}_{\mathrm{future}}$.

To directly optimize communication performance, inspired by multitask learning [17], a multiobjective loss function is defined. It includes the normalized NMSE and an additional spectral efficiency term:

(14) $$L_{\mathrm{NMSE}}=E\left[\frac{∥H_{\mathrm{future}}-{\hat{H}}_{\mathrm{future}}{∥}_{F}^{2}}{∥H_{\mathrm{future}}{∥}_{F}^{2}}\right],$$

LLM4FB was trained according to the NMSE loss alone. To further enhance performance, a combined loss function is proposed:

(15) $$L_{\mathrm{total}}=L_{\mathrm{NMSE}}-\lambda \cdot \frac{\mathrm{detach}(L_{\mathrm{NMSE}})}{\mathrm{detach}(|R|)}\cdot R,$$

where *R* denotes the SE computed using the predicted CSI ${\hat{H}}_{\mathrm{future}}$, and λ is a weighting hyperparameter (set to 0.9 in experiments). The detach(·) operation prevents gradient propagation through this term. Referring to the task-oriented design approach [18], this loss formulation uses SE as a guiding signal to bias the model toward predictions yielding higher SE, while preserving the primary NMSE-driven gradient. In the training procedure, LLM4FB is first trained using the NMSE loss (14), and then LLM4FB+ is obtained by fine-tuning the trained LLM4FB model for 10 additional epochs using the combined loss (15).

Specifically, the term functions as a dynamic coefficient $\alpha_{t}$:

(16) $$\alpha_{t}=\frac{\mathrm{detach}(L_{\mathrm{NMSE}})}{\mathrm{detach}(|R|)}.$$

By multiplying the rate *R* by $\alpha_{t}$, we effectively scale the magnitude of the rate-based loss component to match the current magnitude of the NMSE loss. The detach(·) operation is critical here; it treats $L_{\mathrm{NMSE}}$ and |R| as constants during backpropagation. This prevents the optimizer from manipulating the scaling factor itself to minimize the loss (e.g., by artificially inflating |R| to reduce the weight), ensuring that gradients flow only through the optimization targets.

Consequently, the gradient of the total loss with respect to the network parameters θ is given by

(17) $$\frac{\partial L_{\mathrm{total}}}{\partial \theta }=\frac{\partial L_{\mathrm{NMSE}}}{\partial \theta }-\lambda \cdot \underset{\mathrm{Adaptive}\;\mathrm{Weight}}{\underbrace{{\left(\frac{L_{\mathrm{NMSE}}}{\left|R\right|}\right)}_{\mathrm{fixed}}}}\cdot \frac{\partial R}{\partial \theta }.$$

This formulation ensures that the contribution of the spectral efficiency to the parameter updates is always proportionally aligned with the reconstruction error, allowing for stable joint optimization where the SE maximization task provides a guided auxiliary gradient without overwhelming the primary objective of minimizing NMSE. The parameter λ, thus, acts as a fine-tuning knob for the relative priority of SE, independent of the numerical scale of *R*. The impact of λ will be analyzed in subsequent sections.

## 4. Simulation Results

### 4.1. Simulation Environment and Parameters

The design of wireless datasets has been shown to influence the performance of AI communication systems [19]. An open-source CSI channel prediction dataset is used for the experiments [15,20], which is generated with the QuaDRiGa channel simulator and complies with the 3GPP 38.901 channel standard. This paper consider a single-cell MISO-OFDM system, where the BS is equipped with an $N_{t}=32$ UPA, and the UE has a single antenna. The system bandwidth is 8.64 MHz, comprising 48 resource blocks (RBs), i.e., $N_{\mathrm{sc}}=576$. FDD is adopted, with an uplink center frequency of 2.4 GHz. The model predicts future CSI for $L_{f}=4$ time slots based on historical CSI from $L_{h}=16$ consecutive time slots, with a pilot interval of 0.5 ms. The channel scenario follows the 3GPP urban macro (UMa) non-line-of-sight (NLOS) model. The training, validation, and test sets contain 8000, 1000, and 10,000 samples, respectively, covering UE velocities ranging from 10 km/h to 100 km/h.

To investigate the effect of varying compression ratios (CR) on model performance, CR values are set to {2,4,8,16,32,64}. To ensure a fair comparison, the random seed for the projection matrix generation is fixed.

Several representative baselines are considered for comparison, including the traditional PAD model [21], classical deep learning models (DNN, RNN, LSTM, CNN), and a state-of-the-art Transformer model. As summarized in Table 1, despite the large total parameter count, LLM4FB requires significantly fewer trainable parameters (0.97 M) than other DL models, as only the fine-tuning parameters are updated.

### 4.2. Computational Complexity and Resource Consumption Analysis

To comprehensively evaluate the practical deployment feasibility of LLM4FB, we conduct a detailed analysis of computational complexity, memory consumption, and inference latency for both UE and BS sides. Table 2 presents a comprehensive comparison across all baseline methods.

Params-UE denotes the storage footprint of the projection matrix Φ. All methods except CS-CsiNet employ fixed random projections with zero trainable parameters at the UE side, and CS-CsiNet uses a learned projection matrix, resulting in 0.59 M parameters. CS-CsiNet was originally designed for a compressed feedback task. Now, to adapt it for a prediction task, we have changed the Sigmoid to ReLU and added three linear layers, two LN layers, and ReLU activation functions at the final output. From Table 2, several observations can be made regarding the computational distribution between UE and BS:

All methods maintain identical UE-side complexity (0.045 ms latency, 0.59 M parameters, 10.85 M memory). This confirms that the one-sided architecture successfully decouples the decoder complexity from the UE burden—the terminal only performs a fixed linear projection y=Φx regardless of the BS-side model sophistication. This property is essential for resource-constrained IoT devices and wireless sensors.

LLM4FB achieves a BS-side inference latency of 6.435 ms, which is 6.4 times faster than the Transformer (41.295 ms) while maintaining comparable or better NMSE (0.106 vs. 0.109). This efficiency results from our 6-layer truncated GPT-2 architecture, which prioritizes feature extraction depth over sequence processing length. The 6.435 ms latency remains practical for systems with 0.5 ms pilot intervals, especially considering the substantial performance gain.

Regarding computational resources, LLM4FB requires 480.25 M memory at the BS—higher than traditional models (12.95–263.15 M) but acceptable for modern GPU-equipped base stations. The computational cost of 0.93 GFLOPs is lower than CNN (4.83 GFLOPs). More importantly, LLM4FB (PEFT) reduces NMSE by 45.6% compared to CS-CsiNet (0.106 vs. 0.195), demonstrating that the memory overhead is justified by the performance improvement.

Comparing PEFT with full-parameter training shows that while full training achieves slightly better NMSE (0.087 vs. 0.106), the PEFT strategy requires only 1.57 M trainable parameters and maintains identical inference cost. This validates that the pre-trained LLM already contains sufficient structural knowledge for CSI reconstruction, requiring only minimal fine-tuning of normalization layers and embeddings to adapt to the wireless domain.

### 4.3. Performance Evaluation

Table 3 and Table 4 compare the NMSE and SE performance under varying compression ratios at SNR of 10 dB. Several observations can be drawn.

Regarding NMSE, it can be observed that as the compression ratio increases from 2 to 64, the prediction error of all methods inevitably rises due to information blur. However, LLM4FB consistently achieves the lowest NMSE across all compression ratios, indicating its excellence in CSI reconstruction prediction. Meanwhile, under high compression scenarios (e.g., CR = 64), traditional deep learning methods such as CNN and DNN experience significant performance degradation, whereas LLM4FB maintains relatively low error. This robustness is attributed to LLM’s powerful contextual reasoning capability, enabling it to infer and recover missing channel details from extremely sparse observations. For example, at CR = 8, LLM4FB achieves an NMSE of 0.144, comparable to the best performing Transformer baseline, while using significantly fewer trainable parameters.

In terms of SE, the multiobjective optimized variant, LLM4FB+, exhibits the best overall performance. Although its NMSE is marginally higher than that of the standard LLM4FB in some cases, it achieves the highest SE across all tested compression ratios. This phenomenon highlights that minimizing the mean squared error does not always translate to maximizing the communication rate, as NMSE weights all channel elements uniformly, whereas SE is more sensitive to the accuracy of the dominant eigenmodes used for beamforming. By directly adding SE to the loss function, LLM4FB+ is able to optimize towards the channel features that are most important for improving the downlink rate. At a compression ratio of CR = 8, the model achieves a spectral efficiency of 8.036 bps/Hz, which is about 2.6% higher than the Transformer baseline. This result indicates that the joint optimization approach works as expected.

**Figure 2:**
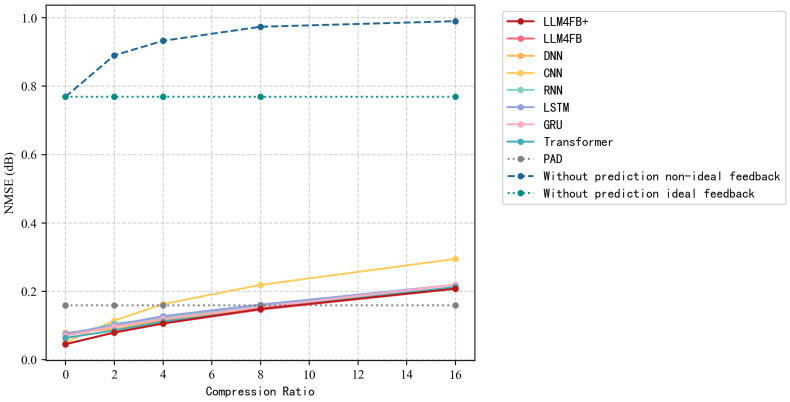
NMSE under different compression ratios (SNR = 10 dB).

**Figure 3:**
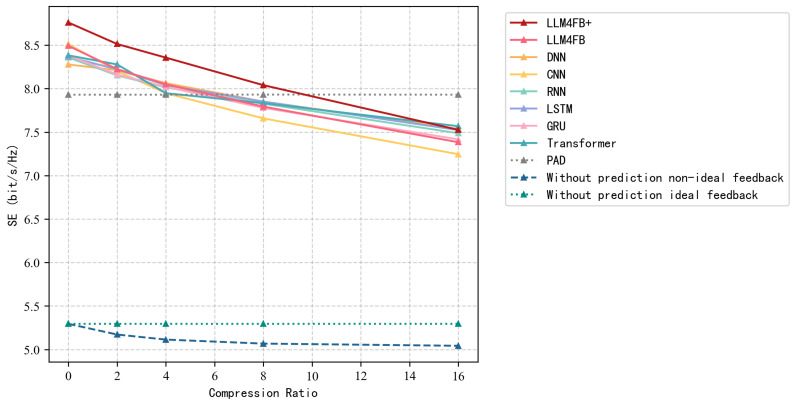
Comparison of spectral efficiency under different compression ratios (SNR = 10 dB).

Figure 2 and Figure 3 illustrate the NMSE and SE performance under varying compression ratios. It is observed that as the compression ratio increases, the NMSE of all methods increases, whereas the SE decreases. LLM4FB and LLM4FB+ consistently outperform the other baseline methods. In scenarios with low compression ratios, the improvement of SE is clearly noticeable.

**Figure 4:**
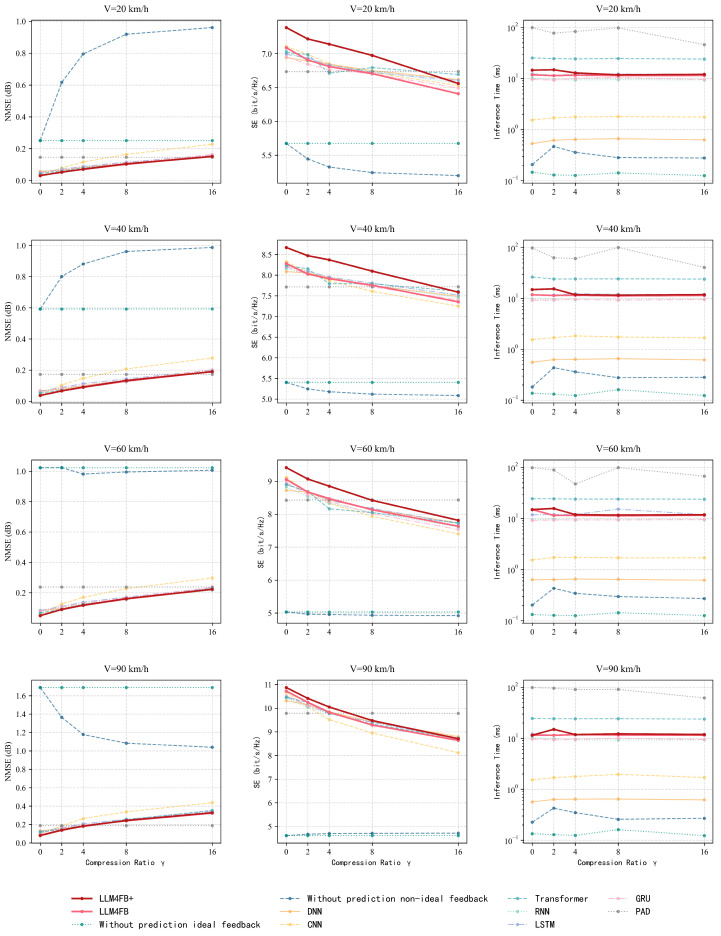
NMSE and spectral efficiency performance under different UE speeds (CR = 8, SNR = 10 dB).

Figure 4 shows the performance of models’ behavior under various UE speeds, from 20 km/h up to 90 km/h. As the user speed increases, the Doppler spread widens, leading to faster temporal variations in the channel impulse response. The performance of all feedback schemes degrades. Nevertheless, LLM4FB and LLM4FB+ demonstrate superior resilience compared to the baselines. Even at high speeds (e.g., 90 km/h), where the channel coherence time is short, our method maintains a significant performance advantage over conventional RNN and LSTM models. This indicates that the pre-trained LLM is able to learn the channel’s temporal patterns to make accurate predictions even for highly dynamic environments.

**Figure 5:**
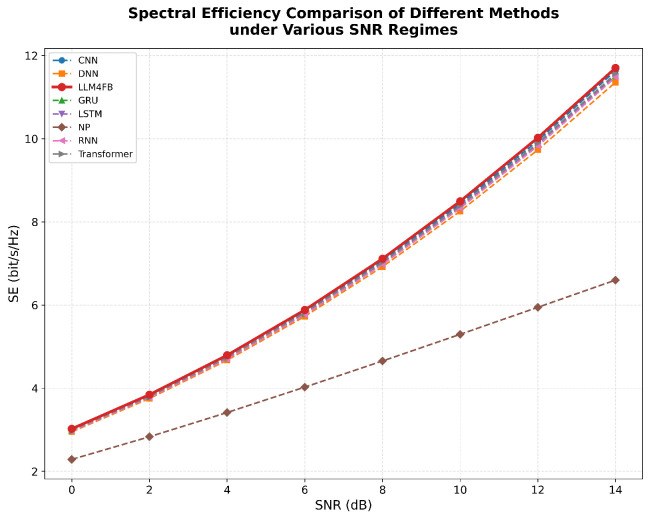
Spectral efficiency comparison under different SNR levels.

Figure 5 illustrates the spectral efficiency performance across varying SNR conditions. As expected, SE increases with higher SNR for all methods due to improved channel quality. Notably, LLM4FB maintains a consistent performance advantage over baseline methods across the entire SNR range. At low SNR (0 dB), the gap is more pronounced, demonstrating the robustness of the LLM-based denoising capability. This validates that our framework achieves robust performance under diverse channel conditions.

### 4.4. Impact of λ on Performance Trade-Offs and Scenario Generalization

To investigate the effect of the multiobjective weight parameter λ in Equation (15) and validate the robustness of LLM4FB across different propagation environments, we conduct ablation experiments across different compression ratios. The parameter λ controls the trade-off between NMSE minimization and SE maximization. To further validate the generalization capability of our framework, we use the pre-trained base model originally trained on the UMa scenario and fine-tune it on the UMi (urban micro) scenario with different λ values. This cross-scenario fine-tuning strategy allows us to verify both the optimal λ range and the model’s adaptability to diverse channel conditions. Table 5 presents the NMSE and SE performance under various λ values ranging from 0 to 2.0.

Table 5 presents the ablation study results for the multiobjective weight parameter λ defined in Equation (15), evaluated across compression ratios from γ=0 to γ=64 at SNR = 10 dB.

From the experimental results, we observe that for PEFT-based models, λ values in the range 0.5–1.0 achieve the best trade-off between NMSE and SE. When λ=0 (pure NMSE optimization), the model achieves the lowest reconstruction error but yields suboptimal SE. For example, at γ=8, the NMSE is 0.143 but SE reaches only 7.782 bps/Hz. As λ increases to 0.9, the NMSE slightly degrades to 0.146, while SE improves significantly to 7.969 bps/Hz—a 2.4% gain. This demonstrates that minimizing NMSE does not necessarily maximize communication rate, as SE is more sensitive to the accuracy of dominant channel eigenmodes used in beamforming.

For full-parameter training models, the performance exhibits less sensitivity to λ variations. Even at λ=2.0, the NMSE degradation remains within 3% compared to λ=0. This indicates that models with sufficient capacity can simultaneously optimize both objectives without severe performance trade-offs. However, excessively large λ values (>1.0) provide diminishing returns and may occasionally degrade NMSE, particularly under high compression ratios.

The sensitivity to λ also varies with compression ratio. At low compression (γ≤4), NMSE variations across different λ values are minimal (<3%), suggesting that abundant feedback information enables easy satisfaction of both objectives. In contrast, at high compression (γ≥32), the choice of λ becomes more critical, with NMSE variations up to 5%. This indicates that careful hyperparameter tuning is essential in resource-constrained scenarios.

Based on these observations, we adopt λ=0.9 for the LLM4FB+ variant in our main experiments, which provides a practical balance between reconstruction accuracy and spectral efficiency.

### 4.5. Model Architecture and Pre-Training Benefits

To address the question of whether performance gains stem from the GPT-2 architecture itself or from leveraging pre-trained weights, Table 6 presents controlled ablation experiments that isolate these two factors.

We construct four variants under identical conditions: (1) Transformer baseline with standard architecture trained from scratch; (2) GPT-2 architecture with random initialization and full-parameter training; (3) GPT-2 with random initialization but only LN layers trainable; (4) our proposed LLM4FB with pre-trained GPT-2 weights and only LN layers fine-tuned.

The results reveal several findings. Comparing GPT-2 (Scratch + Full) with the Transformer baseline shows that the GPT-2 architecture itself provides substantial benefits even without pre-training. At γ=0, NMSE improves from 0.064 to 0.047, and at γ=8, from 0.146 to 0.109. This 25–26% reduction demonstrates that GPT-2’s design—including its residual connections, layer normalization placement, and attention patterns—is inherently more suitable for CSI reconstruction.

The failure of GPT-2 (Scratch + PEFT) is particularly instructive. With only LN layers trainable from random initialization, the model performs worse than even the Transformer baseline at high compression (NMSE 0.562 vs. 0.494 at γ=64). This indicates that fine-tuning only 0.97 M parameters out of 85.23 M total is insufficient when starting from random weights. The model simply cannot learn meaningful channel representations with such limited trainable capacity.

LLM4FB (pre-trained + PEFT) achieves the best performance across all compression ratios despite having the same training configuration as GPT-2 (Scratch + PEFT). At γ=8, NMSE is 0.145 compared to 0.156 for the randomly initialized version, representing a 7% improvement. At γ=64, the gap widens further, with NMSE of 0.464 compared to 0.562 for the randomly initialized variant, corresponding to a 17% improvement. This comparison directly demonstrates the value of pre-trained weights: they provide a strong initialization that enables successful adaptation with minimal parameter updates.

These results establish that LLM4FB’s effectiveness arises from three factors working together: a well-designed architecture optimized for sequential modeling, pre-trained weights encoding general temporal patterns, and an efficient fine-tuning strategy that adapts only the normalization layers.

### 4.6. Fine-Tuning Strategy Comparison: LN-Only and LN + PE

To determine the optimal parameter-efficient fine-tuning configuration, we compare two strategies: fine-tuning only layer normalization (LN) parameters (0.97 M trainable) versus jointly fine-tuning LN and positional embedding (PE) parameters (1.76 M trainable). Both strategies maintain significantly lower trainable parameter counts compared to full fine-tuning (85.23 M), but differ in which components are updated during adaptation. Table 7 presents the performance comparison across compression ratios from γ=0 to γ=64.

Table 7 presents the performance comparison between two parameter-efficient fine-tuning configurations: fine-tuning only layer normalization (LN) parameters (0.97 M trainable) versus jointly fine-tuning LN and positional embedding (PE) parameters (1.76 M trainable).

The results reveal that both strategies achieve nearly identical performance across all compression ratios. At γ=0, the NMSE difference is merely 0.001 (0.044 vs. 0.045), representing a negligible 2.3% variation. Similarly, SE values are 8.494 and 8.492 bps/Hz, respectively—effectively identical within measurement precision. This pattern persists across the entire compression range: at γ=8, NMSE values are 0.145 vs. 0.146 (0.7% difference), and at γ=64, they are 0.464 vs. 0.465 (0.2% difference). The SE performance exhibits comparable consistency, with differences typically below 0.5%.

The minimal performance gap between the two configurations indicates that fine-tuning only layer normalization parameters is sufficient for effective domain adaptation. Layer normalization controls activation distributions through scale (γ) and shift (β) parameters:

(18) $$\mathrm{LN}\left(x\right)=\gamma \cdot \frac{x-\mu }{\sqrt{\sigma^{2}+\epsilon }}+\beta ,$$

By adjusting these parameters, the model recalibrates pre-trained feature representations to match wireless channel statistics without modifying core attention mechanisms. The positional embeddings, which encode temporal ordering information, appear largely redundant in this task—the self-attention mechanism already captures temporal dependencies through learned attention weights during pre-training.

The practical implication is significant: adding 0.79 M more trainable parameters provides no measurable performance benefit. This validates our design choice of fine-tuning only LN layers as the default configuration for LLM4FB. The LN-only strategy offers three advantages: (1) 45% fewer trainable parameters, reducing training memory requirements; (2) faster convergence due to smaller optimization space; (3) implicit regularization that may prevent overfitting in data-limited scenarios.

### 4.7. Denoising Capability Analysis Under High Compression

A critical concern in one-sided feedback with high compression ratios is the severe noise introduced by the pseudoinverse reconstruction. To validate the LLM’s denoising capability, we analyze the reconstruction quality at different processing stages across various compression ratios.

Table 8 quantifies the reconstruction quality at three critical stages: (1) the initial pseudoinverse estimate ${\hat{H}}_{\mathrm{init}}$ for historical CSI, (2) a naive baseline that repeats the last historical time slot as future prediction (NMSE = 0.769 across all CRs), and (3) the final LLM-enhanced output ${\hat{H}}_{\mathrm{future}}$. Several observations validate the LLM’s robust denoising and prediction capability.

**Figure 6:**
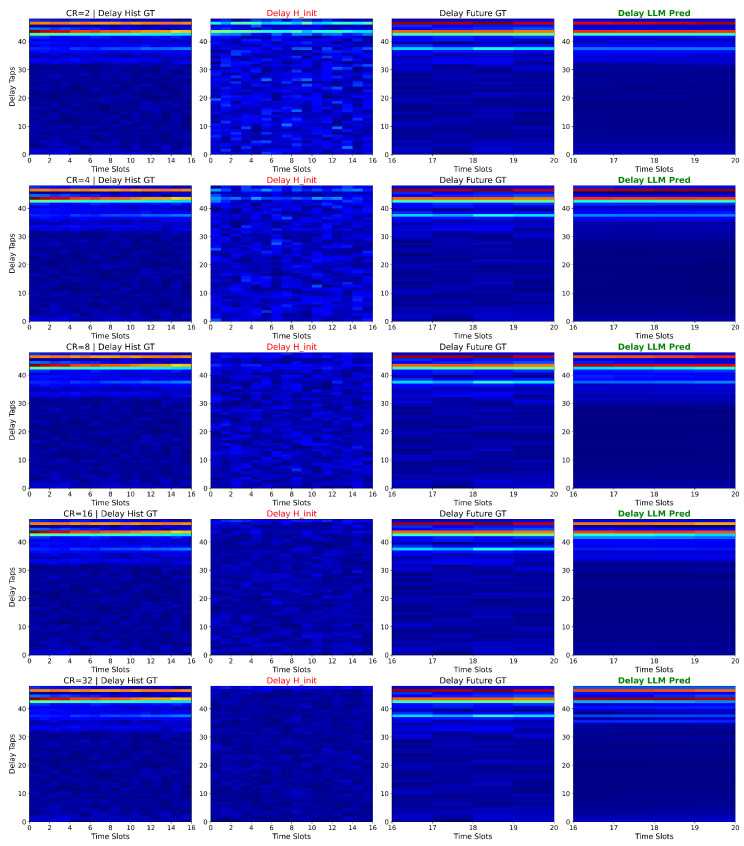
Visualization of delay-domain CSI at different compression ratios and processing stages. The column titled in red (Delay H_init) represents the initial coarse recovery results, while the column titled in green (Delay LLM Pred) showcases the final reconstruction results of the proposed LLM4FB model.

Figure 6 visualizes the delay-domain CSI across different compression ratios and processing stages. Each row corresponds to a specific CR (γ∈{2,4,8,16,32,64}), and the four columns show the following: (1) Delay Hist GT—ground truth historical CSI in delay domain, (2) Delay 4 Init—pseudoinverse reconstruction ${\hat{H}}_{\mathrm{init}}$ transformed to delay domain, (3) Delay Future GT—ground truth future CSI, and (4) Delay LLM Pred—LLM prediction ${\hat{H}}_{\mathrm{future}}$ in delay domain.

These results demonstrate that even under extreme compression (e.g., CR = 64), where the initial pseudoinverse estimate is highly noisy (NMSE = 0.985), the LLM is able to reduce the error. This indicates that the LLM denoises the corrupted input by leveraging learned temporal patterns.

## 5. Conclusions

In this paper, we proposed LLM4FB, a novel one-sided CSI feedback and prediction framework that leverages a pre-trained LLM at the BS. The use of the LLM allows most of the computational load to be handled at the BS, which makes the approach easier to deploy in practice and keeps the UE side implementation lightweight. With this design, the system can reconstruct and predict CSI accurately without adding extra complexity to the UE. This paper also adopts the approach of multitask learning and has developed a loss function that both targets NMSE and SE. Experimental results show that the framework performs well under different ratios and mobility conditions, providing a practical way to reduce UE overhead while improving CSI prediction accuracy. This lightweight characteristic makes LLM4FB highly applicable to intelligent sensor interface systems and massive IoT connectivity, bridging the gap between advanced AI models and resource-limited sensor hardware.

## Figures and Tables

**Table 1:** Comparison of total and trainable parameters (in millions) for each algorithm at CR = 8.

Algorithm	Total Parameters	Trainable Parameters
PAD	0	0
RNN	3.10	2.51
LSTM	10.18	9.59
CNN	3.73	3.14
Transformer	12.35	11.76
DNN	2.92	2.35
CS-CsiNet	2.36	2.36
**LLM4FB**	**85.23**	**0.97**

Note: Bold font indicates the proposed LLM4FB algorithm.

**Table 2:** Comprehensive comparison of computational resources and latency for UE and BS (CR = 4).

Model	NMSE	Latency-UE	Latency-BS	Params-UE	Params-BS	FLOPs-UE	FLOPs-BS	Memory-UE	Memory-BS
		**(ms)**	**(ms)**	**(M)**	**(M)**	**(G)**	**(G)**	**(M)**	**(M)**
Direct Feedback	0.932	0.045	0.045	0.59	0.59	<0.01	<0.01	10.85	10.85
DNN	0.114	0.045	0.305	0.59	2.93	<0.01	0.01	10.85	12.95
RNN	0.131	0.045	4.255	0.59	3.10	<0.01	0.10	10.85	32.75
LSTM	0.129	0.045	5.145	0.59	10.19	<0.01	0.37	10.85	81.95
GRU	0.120	0.045	4.785	0.59	7.83	<0.01	0.28	10.85	61.35
CNN	0.162	0.045	1.085	0.59	3.74	<0.01	4.83	10.85	263.15
Transformer	0.109	0.045	41.295	0.59	12.36	<0.01	0.21	10.85	82.65
CS-CsiNet	0.195	0.045	1.095	0.59	4.14	<0.01	0.33	10.85	246.55
LLM4FB (all parameters)	0.087	0.045	6.435	0.59	1.57	<0.01	0.93	10.85	480.25
**LLM4FB (PEFT)**	0.106	0.045	6.435	0.59	1.57	<0.01	0.93	10.85	480.25

Note: Bold font indicates the proposed LLM4FB algorithm. The LLM4FB (all parameters) row represents the model trained with full-parameter fine-tuning, while LLM4FB (PEFT) denotes the parameter-efficient fine-tuning approach.

**Table 3:** NMSE performance comparison of different methods.

Method	CR = 0	CR = 2	CR = 4	CR = 8	CR = 16	CR = 32	CR = 64
CS-CsiNet	0.197 ± 0.002	0.200 ± 0.002	0.216 ± 0.001	0.219 ± 0.003	0.284 ± 0.004	0.469 ± 0.004	0.663 ± 0.003
DNN	0.079 ± 0.003	0.091 ± 0.001	0.116 ± 0.001	0.149 ± 0.004	0.209 ± 0.001	0.305 ± 0.003	0.472 ± 0.001
CNN	0.051 ± 0.002	0.115 ± 0.004	0.162 ± 0.002	0.220 ± 0.001	0.296 ± 0.003	0.388 ± 0.001	0.530 ± 0.002
RNN	0.076 ± 0.002	0.106 ± 0.001	0.123 ± 0.003	0.156 ± 0.002	0.212 ± 0.002	**0.303 ± 0.004**	0.471 ± 0.002
LSTM	0.077 ± 0.002	0.101 ± 0.003	0.127 ± 0.003	0.162 ± 0.002	0.220 ± 0.001	0.328 ± 0.003	0.502 ± 0.004
GRU	0.071 ± 0.004	0.097 ± 0.002	0.118 ± 0.003	0.155 ± 0.003	0.220 ± 0.001	0.321 ± 0.003	0.488 ± 0.001
Transformer	0.064 ± 0.002	0.085 ± 0.003	0.113 ± 0.001	0.146 ± 0.004	0.214 ± 0.003	0.309 ± 0.003	0.489 ± 0.002
**LLM4FB**	0.044 ± 0.003	**0.079 ± 0.003**	**0.106 ± 0.001**	**0.144 ± 0.002**	0.208 ± 0.003	0.304 ± 0.004	**0.461 ± 0.003**
**LLM4FB+**	**0.042 ± 0.003**	0.080 ± 0.002	0.107 ± 0.003	0.149 ± 0.004	**0.207 ± 0.003**	**0.303 ± 0.004**	0.464 ± 0.003

Note: Bold font indicates the proposed LLM4FB algorithm. The best performance for each compression ratio is highlighted in bold.

**Table 4:** Rate performance comparison of different methods.

Method	CR = 0	CR = 2	CR = 4	CR = 8	CR = 16	CR = 32	CR = 64
CS-CsiNet	7.643 ± 0.023	7.531 ± 0.020	7.524 ± 0.026	7.448 ± 0.016	6.979 ± 0.018	6.082 ± 0.038	5.383 ± 0.038
DNN	8.265 ± 0.014	8.206 ± 0.011	8.058 ± 0.030	7.846 ± 0.037	7.531 ± 0.020	**7.052 ± 0.026**	**6.372 ± 0.013**
CNN	8.453 ± 0.036	8.198 ± 0.030	7.943 ± 0.037	7.653 ± 0.037	7.242 ± 0.038	6.777 ± 0.035	6.178 ± 0.031
RNN	8.356 ± 0.014	8.149 ± 0.011	8.010 ± 0.022	7.823 ± 0.037	7.483 ± 0.031	7.026 ± 0.032	6.335 ± 0.038
LSTM	8.358 ± 0.010	8.218 ± 0.028	8.037 ± 0.035	7.847 ± 0.013	7.520 ± 0.031	7.008 ± 0.019	6.294 ± 0.033
GRU	8.290 ± 0.038	8.158 ± 0.016	8.017 ± 0.022	7.775 ± 0.032	7.413 ± 0.011	6.953 ± 0.029	6.329 ± 0.011
Transformer	8.355 ± 0.024	8.274 ± 0.017	7.951 ± 0.022	7.829 ± 0.016	**7.562 ± 0.012**	7.048 ± 0.036	6.268 ± 0.033
**LLM4FB**	8.506 ± 0.025	8.227 ± 0.034	8.054 ± 0.023	7.795 ± 0.033	7.386 ± 0.022	6.891 ± 0.036	6.292 ± 0.034
**LLM4FB+**	**8.603 ± 0.017**	**8.509 ± 0.037**	**8.349 ± 0.038**	**8.036 ± 0.027**	7.528 ± 0.036	6.976 ± 0.018	6.278 ± 0.019

Note: Bold font indicates the proposed LLM4FB algorithm. The best performance for each compression ratio is highlighted in bold.

**Table 5:** NMSE and SE performance under different λ values across compression ratios (SNR = 10 dB).

Model Configuration	γ=0		γ=2		γ=4		γ=8		γ=16		γ=32		γ=64	
	NMSE	SE	NMSE	SE	NMSE	SE	NMSE	SE	NMSE	SE	NMSE	SE	NMSE	SE
LLM4FB (PEFT, λ=0)	0.043	8.495	0.076	8.253	0.103	8.023	0.143	7.782	0.206	7.394	0.296	6.943	0.462	6.311
LLM4FB+ (PEFT, λ=0.001)	0.044	8.499	0.076	8.257	0.104	8.041	0.143	7.791	0.206	7.398	0.297	6.935	0.461	6.311
LLM4FB+ (PEFT, λ=0.01)	0.043	8.497	0.076	8.254	0.103	8.049	0.143	7.799	0.206	7.409	0.297	6.964	0.462	6.312
LLM4FB+ (PEFT, λ=0.1)	0.043	8.541	0.076	8.308	0.104	8.075	0.143	7.853	0.206	7.456	0.298	6.976	0.465	6.321
LLM4FB+ (PEFT, λ=0.5)	0.044	8.631	0.077	8.417	0.106	8.255	0.145	7.966	0.208	7.492	0.300	6.964	0.466	6.334
LLM4FB+ (PEFT, λ=0.9)	0.045	8.779	0.078	8.516	0.106	8.281	0.146	7.969	0.210	7.527	0.300	6.994	0.471	6.340
LLM4FB+ (PEFT, λ=1.0)	0.046	8.788	0.078	8.554	0.108	8.316	0.148	7.967	0.212	7.437	0.301	6.991	0.467	6.330
LLM4FB+ (PEFT, λ=2.0)	0.047	8.850	0.081	8.643	0.110	8.268	0.154	8.058	0.210	7.460	0.300	6.963	0.469	6.312
LLM4FB (all parameters, λ=0)	0.046	8.438	0.071	8.328	0.084	8.174	0.109	8.013	0.167	7.766	0.255	7.268	0.433	6.461
LLM4FB+ (all parameters, λ=0.001)	0.046	8.444	0.071	8.331	0.084	8.173	0.109	8.012	0.166	7.763	0.256	7.273	0.435	6.451
LLM4FB+ (all parameters, λ=0.01)	0.046	8.472	0.071	8.334	0.084	8.174	0.109	8.021	0.166	7.770	0.255	7.270	0.434	6.450
LLM4FB+ (all parameters, λ=0.1)	0.046	8.510	0.072	8.361	0.084	8.207	0.109	8.052	0.168	7.785	0.258	7.265	0.438	6.449
LLM4FB+ (all parameters, λ=0.5)	0.046	8.647	0.072	8.473	0.085	8.300	0.112	8.090	0.170	7.844	0.258	7.318	0.442	6.450
LLM4FB+ (all parameters, λ=0.9)	0.048	8.733	0.073	8.508	0.086	8.373	0.111	8.185	0.172	7.881	0.261	7.324	0.443	6.452
LLM4FB+ (all parameters, λ=1.0)	0.048	8.717	0.074	8.562	0.087	8.341	0.112	8.181	0.171	7.876	0.261	7.293	0.443	6.453
LLM4FB+ (all parameters, λ=2.0)	0.050	8.833	0.076	8.555	0.090	8.340	0.114	8.219	0.175	7.890	0.265	7.306	0.445	6.451

**Table 6:** Ablation study disentangling architecture capacity from pre-training benefits (SNR = 10 dB).

Model Variant	Initialization	Training Strategy	NMSE (γ=0)	NMSE (γ=8)	NMSE (γ=64)	Attribution
Transformer (Baseline)	Random	Full Parameters	0.064	0.146	0.494	Baseline
GPT-2 (Scratch + Full)	Random	Full Parameters	0.047	0.109	0.440	Architecture Capacity
GPT-2 (Scratch + PEFT)	Random	PEFT Only	0.051	0.156	0.562	PEFT Without Pre-training
**LLM4FB (Pre-trained + PEFT)**	**Pre-trained**	**PEFT Only**	**0.044**	**0.145**	**0.464**	**Architecture + Pre-training**

Note: Bold font indicates the proposed LLM4FB model. PEFT: Parameter-Efficient Fine-Tuning.

**Table 7:** Performance comparison between LN-only and LN + PE fine-tuning strategies (SNR = 10 dB, UMa scenario).

Strategy	Trainable Params (M)	γ=0		γ=2		γ=4		γ=8		γ=16		γ=32		γ=64	
		NMSE	SE	NMSE	SE	NMSE	SE	NMSE	SE	NMSE	SE	NMSE	SE	NMSE	SE
**LN Only**	**0.97**	**0.044**	**8.494**	**0.076**	**8.256**	**0.106**	8.038	**0.145**	**7.777**	**0.205**	**7.436**	0.300	**6.952**	**0.464**	6.280
LN + PE	1.76	0.045	8.492	0.077	8.252	0.106	**8.055**	0.146	7.758	0.209	7.399	**0.299**	6.936	0.465	**6.290**

Note: Bold font indicates the better performance between the two strategies. PE: Positional Embedding.

**Table 8:** Reconstruction quality (NMSE) at different stages under varying compression ratios (SNR = 10 dB).

CR	2	4	8	16	32	64
Pseudoinverse ${\hat{H}}_{\mathrm{init}}$ (past)	0.500	0.743	0.877	0.940	0.969	0.985
Ground Truth Repetition (future)	0.769 (constant baseline)					
LLM4FB Output ${\hat{H}}_{\mathrm{future}}$	0.077	0.106	0.146	0.209	0.301	0.464
NMSE Reduction (past → output)	84.5%	85.7%	83.4%	77.8%	68.9%	52.8%
NMSE Reduction (repetition → output)	90.0%	86.2%	81.0%	72.8%	60.8%	39.6%

## Data Availability

The data presented in this study are available on request from the corresponding author.

## Abbreviations

The following abbreviations are used in this manuscript:

$N_{t}$	Number of transmit antennas at BS
$N_{h},N_{v}$	Number of antennas in horizontal and vertical dimensions
$N_{c}$	Number of scattering clusters
$N_{path}$	Number of propagation paths per cluster
$N_{\mathrm{sc}}$	Number of active subcarriers
$N_{\mathrm{delay}}$	Dimension of delay-domain representation
$L_{h}$	Number of historical time slots
$L_{f}$	Number of future time slots to predict
*P*	Patch size for tokenization
*S*	Number of patches after partitioning
$d_{\mathrm{model}}$	Hidden dimension of LLM
$d_{k}$	Dimension of key vectors in attention mechanism
$L_{\mathrm{layer}}$	Number of layers in LLM backbone
*M*	Dimension of compressed CSI representation
$h_{k,t}$	Channel vector at subcarrier *k* and time *t*
$H_{\mathrm{past}}$	Historical CSI tensor
$H_{\mathrm{future}}$	Future CSI tensor to be predicted
${\hat{H}}_{\mathrm{init}}$	Initial pseudoinverse reconstruction
${\hat{H}}_{\mathrm{future}}$	Predicted future CSI
Φ	Random projection matrix
$\Phi^{†}$	Moore–Penrose pseudoinverse of Φ
$y_{i}$	Compressed representation for antenna *i*
$w_{k,t}$	Pre-coding vector at subcarrier *k* and time *t*
$W_{e}$	Learnable projection matrix for patch embedding
$E_{j}$	Embedded representation of patch *j*
$Z_{\mathrm{LLM}}$	Output features from LLM backbone
$\alpha_{c,l}$	Complex gain of path *l* in cluster *c*
$\tau_{c,l}$	Delay of path *l* in cluster *c*
$\phi_{c,l},\theta_{c,l}$	Azimuth and elevation angles
$f_{D,c,l}$	Doppler shift of path *l* in cluster *c*
*v*	UE speed
$f_{c}$	Carrier frequency
$\sigma_{n}^{2}$	Noise power
λ	Weight parameter in multiobjective loss
